# fMRI evidence of a hot-cold empathy gap in hypothetical and real aversive choices

**DOI:** 10.3389/fnins.2013.00104

**Published:** 2013-06-10

**Authors:** Min J. Kang, Colin F. Camerer

**Affiliations:** ^1^Humanities and Social Sciences, California Institute of TechnologyPasadena, CA, USA; ^2^Computational and Neural Systems, California Institute of TechnologyPasadena, CA, USA

**Keywords:** hypothetical bias, aversive bads, fMRI, medial prefrontal cortex, amygdala, decision making, neuroeconomics

## Abstract

Hypothetical bias is the common finding that hypothetical monetary values for “goods” are higher than real values. We extend this research to the domain of “bads” such as consumer and household choices made to avoid aversive outcomes (e.g., insurance). Previous evidence of hot-cold empathy gaps suggest food disgust is likely to be strongly underestimated in hypothetical (cold) choice. Depending on relative underestimation of food disgust and pain of spending, the hypothetical bias for aversive bad scan go in the typical direction for goods, disappear, or reverse in sign. We find that the bias is reversed in sign—subjects pay more to avoid bads when choice is real. fMRI shows that real choice more strongly activates striatum and medial prefrontal cortex (reward regions) and shows distinct activity in insula and amygdala (disgust and fear regions). The neural findings suggest ways to exogeneously manipulate or record brain activity in order to create better forecasts of actual consumer choice.

## Introduction

Real choices are binding consequential commitments to a course of action, like undergoing surgery or putting a down payment on a house. Researchers in all social sciences seek to understand how real choices are made. However, in studying decisions, scientists and policy makers often have to settle for measuring hypothetical statements about what people *would* choose, rather than observing what people actually *do* choose.

In marketing research, for example, hypothetical surveys are used to forecast sales of existing products, to test new products by asking consumers what they would buy, and to evaluate promotions (Silk and Urban, 1978; Urban et al., 1983; Infosino, 1986; Jamieson and Bass, 1989; Green and Srinivasan, 1990; Chandon et al., 2004; Raghubir and Greenleaf, 2006; Schlosser et al., 2006). In public economics and political science, survey data are used to establish the dollar value of goods that are not traded in markets (such as clean air or the prevention of oil spills), and to poll likely voters before an election (Crespi, 1989; Diamond and Hausman, 1994; Carson et al., 1996; Mortimer and Segal, 2008). Hypothetical choices are also necessary in some types of psychology and neuroscience experiments in which measuring real choices is impractical or unethical, especially in the domain of distressing moral choices (Greene et al., 2001, 2004; Kühberger et al., 2002; Hariri et al., 2006; Monterosso et al., 2007).

The reliance on hypothetical choice data presumes either that hypothetical choices are a good and legitimate way to forecast real choices, or that there is some knowable relationship between the two types of choices, such that the hypothetical data can be adjusted to forecast real choice data accurately.

However, many studies in behavioral economics have shown a substantial, systematic gap: typically, hypothetical valuations are greater than real valuations (Cummings et al., 1995; Johannesson et al., 1998; List and Gallet, 2001; Ariely and Wertenbroch, 2002; Little and Berrens, 2004; Murphy et al., 2005; Blumenschein et al., 2007; Tanner and Carlson, 2009). To remedy this typical upward “Yes” bias, in marketing research conjoint analysis using hypothetical preference data has been extended and improved through hybrid incentive-aligned methods in which an inferred choice will be implemented for real (Ding et al., 2005; Ding, 2007; Dong et al., 2010). In the moral domain, FeldmanHall et al. (2012a,b) find that subjects say they will sacrifice more money to spare others' from mild electrical shocks than they actually do when the shocks are real.

However, most of these studies comparing hypothetical and real choices used *appetitive goods*, that is, goods to which people assign a positive value. Our paper is only the second to compare hypothetical and real economic valuations of *aversive “bads”* (following FeldmanHall et al., 2012b). We do so using functional magnetic resonance imaging (fMRI). The main goal is to see whether there is distinct neural valuation during hypothetical and real choices about aversive bads, with the hope that knowing where those valuations are encoded would lead to new predictions.

In our choice paradigm, subjects choose how much they would pay to *avoid* having to eat a food that most people find unpleasant (such as pigs' feet, canned oyster, or a large dollop of spicy wasabi; see Table A1 and Plassmann et al., 2010). Eating these foods is, of course, not as dramatic as some naturally-occurring bads that consumers must spend money or effort to avoid, such as regular colonoscopy screenings or protecting against identity theft. However, the advantage of using bad foods is that consenting subjects can actually make these unpleasant real choices in a lab environment. That is, at the end of the experiment they actually eat one food if they don't pay enough to avoid it. Having real choices is crucial, of course, for the comparative study of real and hypothetical choices. It is expected that initial clues from fMRI during unpleasant decisions about bad foods will provide some guidance regarding the neural valuation of more dramatic and unpleasant aversive experiences.

Hypothetical measures *are* often used to judge the value of *aversive bads*. One category of bads is environmental damage (Carson et al., 2003; Loureiro et al., 2009; von Stackelberg and Hammitt, 2009; Martin-Ortega et al., 2011). Another category includes a public good that benefits society but harms a host location, such as locating a prison or a toxic waste dump. In studies of medical decision making, patients are often asked to choose between hypothetical medical treatments that could involve serious side effects (Silvestri et al., 1998; Levy and Baron, 2005), or to express valuations of those procedures in numerical terms such as quality-adjusted life years (QALYs) (Zeckhauser and Shepard, 1976). In all these cases, it is difficult or impossible to compare hypothetical choices with real ones.

Why are aversive choices interesting? Many everyday choices require paying—money, as well as effort—to avoid unpleasant and harmful events. Consider insurance. The purchase of a car or earthquake insurance policy, an AppleCare service package, or an alarm system does not have any appetitive value, per se; instead, it is a payment to avoid future aversive events (similar to paying to avoid unpleasant food). Similarly, going to the doctor or dentist, taking medicines with side effects, and dieting and exercise, are (typically) aversive choices to prevent even worse future outcomes. Political campaigns also use marketing tactics, to persuade voters to accept aversive tradeoffs (such as raising taxes to eliminate California's deficit, or cutting pensions in Greece). Finally, many household purchases might be appetitive for one household member but aversive for others (e.g., one spouse suffering through a summer action movie, or a teenager dragged to a bed-and-breakfast with her parents). If the unfortunate spouse or teenager misforecasts how aversive the activity will really be, during hypothetical planning, then our study could shed some light on how to market such mixed-valence family activities. Our results would have most direct application in situations where decisions are made to avoid viscerally unpleasant outcomes like pain. It remains to be tested whether they can generalize to less visceral decisions like purchasing an extended warranty. We hope that the present study inspires future research that extends to less visceral domains of aversive choice.

Using fMRI in our study establishes tentative findings about whether there are differences in neural circuitry in making hypothetical and real choices involving aversive bads. Our data extend the two previous fMRI studies on this topic, which used appetitive consumer *goods* (Kang et al., 2011) and distressing moral choice (FeldmanHall et al., 2012b). Our paper also adds to emerging literature on consumer neuroscience (Knutson et al., 2007; Plassmann et al., 2011, 2012; Yoon et al., 2012).

Behaviorally, comparing bads and goods could also illuminate the general mechanisms which create differences in hypothetical and real choices. Note that evaluating goods requires a comparison of the utility from a positively-valued appetitive good with an aversive payment of money. A hypothetical bias could result from either overvaluation of appetitive goods, or undervaluation of the distaste of paying money, or both, during hypothetical choice. Studying only appetitive goods cannot discriminate which type of biased evaluation is occurring.

For aversive bads, overly positive hypothetical evaluation (Tanner and Carlson, 2009) leads to underestimation of two different kinds of disutility—disutility from eating aversive foods *and* disutility from paying money. If both are underestimated during hypothetical choice, it is unclear which effect is likely to be more dominant *a priori*, so the difference in hypothetical and real choices is unclear. In fact, there are three possible hypotheses about which effect could predominate and what the sign of the hypothetical-real bias will be.

First, suppose that in hypothetical choice, there is a general underestimation of how bad spending money is (as compared to real payment), and further, that this underestimation is more substantial than the error in the predicted disutility from the consumption of bad food. Then in the aversive bads domain that we study, real willingness-to-pay (WTP) will be lower in magnitude (i.e., closer to zero payment) than hypothetical WTP. This result would unify the findings for appetitive goods and aversive goods; both could then be explained by an insufficient appreciation, in hypothetical situations, for the distasteful spending of money in real choices—the “pain of paying” (Prelec and Loewenstein, 1998). According to this hypothesis 1, dollar values are inflated in hypothetical choice and are deflated toward zero when choices are real (e.g., paying $100 is not too painful when it's not real spending, be it for goods or bads).

Second, an alternative and more plausible hypothesis 2 is that in hypothetical choices, people will underestimate the aversive experience of eating bad foods to a greater extent than they may underestimate the pain of paying. Consider the extreme example of eating a monkey brain (as in the movie “Indiana Jones”) for real. For many people, this would cause an immediate visceral response (e.g., nausea, feeling of disgust). And to be clear, disgust is indeed a visceral, as in physiological, response. For example, Harrison et al. (2010) found that ratings of disgust after watching repulsive food videos led to stronger gastric stomach responses and neural activity in the insula and thalamus.

In contrast, losing an abstract secondary reward, such as money, is likely to elicit less visceral and more cognitive “pain”.^1^ In hypothetical choices that are purely cognitive and have no binding consequences, the brain might make rapid and effortless decisions without fully taking any visceral factors into account. However, in real choices, visceral factors such as disgust are likely to be weighed more heavily, especially for food choices.

The reasoning laid out above is consistent with “hot-cold empathy gaps” (Kühberger et al., 2002). According to Loewenstein (1996, 2005), when making a decision, people underestimate or ignore the effect of visceral factors (generally aversive) such as thirst, fear, and craving for tobacco that are not currently experienced. More specifically, when people are in an affective *cold* state (e.g., not experiencing thirst or craving at the moment), they do not accurately estimate how much such visceral states (*hot* states) will change their preference and behavior, hence the term “hot-cold empathy gap.” For example, smokers who were not having a craving for a cigarette underestimated how much they would value a cigarette when they were later in a high craving state (Sayette et al., 2008). A similar gap has been shown for thirst and for embarrassing public performance (e.g., miming) (Van Boven and Loewenstein, 2003; Loewenstein, 2005; Van Boven et al., 2005), in evaluations of impulsivity of others and social pain (Nordgren et al., 2007, 2011), and in heroin addicts (Badger et al., 2007).

A bigger hot-cold empathy gap for food disgust than for money payment implies the *opposite* of the typical hypothetical > real bias for goods. For real bad-food choices, the aversion to eating a bad food will be strongly adjusted upward and the aversion to paying money will be adjusted upward, by a smaller amount. Real valuations will then be higher than hypothetical valuations (i.e., real WTP > hypothetical WTP), reversing the typical hypothetical bias.

As far as neural activity, we expect that during real choice there will be stronger neural activations in affective areas implicated in disgust and fear processing, such as the insula and amygdala (Whalen, 1998; Craig, 2002, 2009). That is, in this account stronger affective reactions during real choice cause higher bids to avoid eating the foods which are affectively unpleasant. This is an important step because no previous study of hot-cold empathy gaps has compared biological activity in hot and cold states, and shown direct neural evidence consistent with an affective (empathic, or emotional) difference. In addition, based on findings in (Plassmann et al., 2010) we expect higher neural activity in valuation regions such as medial OFC and ventral striatum, when bids are lower. Since these are bids to avoid an unpleasant experience, bidding low means the experience has less negative value.

Note that alternatively, it is possible that affective reactions are only prominent in the real choice when subjects bid a low amount, so they have a high expectation of having to bid the food. That is, in this alternative account, low bids cause stronger affective reactions (rather than the other way around, as in the previous paragraph).

The third and last hypothesis is that the aversion to bad food and the pain of paying are equally underestimated (or not underestimated at all). In this scenario, it is predicted that there will be no significant behavioral difference between real and hypothetical valuations, and fewer neural differences in the two conditions.

The elements of these hypotheses can be mathematically summarized as follows. Let *w* be an individual's WTP to avoid bad food, *x* pain of paying, and *y* disutility from consuming bad food. And let (*w*_*r*_, *x*_*r*_, *y*_*r*_) and (*w*_*h*_, *x*_*h*_, *y*_*h*_) denote the levels of *w*, *x*, *y* in the real and hypothetical conditions, respectively. Suppose that *w*, *x*, and *y* have a functional relationship, *w* = *f*(*x*, *y*).

Generally, a person will be less willing to pay to avoid aversive bads if he feels more pain of paying. Likewise, all other things being equal, he will be more willing to pay to avoid bad food if the disutility from consuming it increases (e.g., he feels more strongly disgusted by it). These relationships can be mathematically expressed as:

(1) $$\frac{\partial f}{\partial x}<0,\;\frac{\partial f}{\partial y}>0$$

Typical consumers are overly optimistic (Weinstein, 1980, 1987, 1989; Tanner and Carlson, 2009) and are thus expected, in hypothetical situations, to misforecast (i.e., underestimate) the real pain of paying as well as the real badness of consuming bad food. This can be expressed as:

(2) $$dx\equiv x_{h}-x_{r}<0\;dy\equiv y_{h}-y_{r}<0$$

where *dx* and *dy* denote the changes in *x* and *y* between the hypothetical and real conditions.

Given the above formalism, the change in hypothetical and real WTP, denoted *dw*, can be written as: 
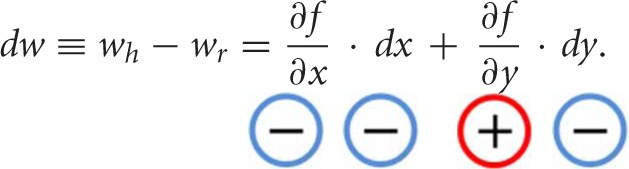
 Note the signs of the terms on the right-hand side from Equations (1, 2); the sign of *dw* (e.g., the relative size of hypothetical and real WTP) is determined by the relative size of the terms on the right as summarized in Table 1.

**Table 1:** **Mathematical summary of the three hypotheses**.

	**Relative size of changes**	**Effect**
Hypothesis 1	$\left|\frac{\partial f}{\partial x}\;\cdot \;dx\right|>\left|\frac{\partial f}{\partial y}\;\cdot \;dy\right|$	*w*_*h*_ > *w*_*r*_
Hypothesis 2	$\left|\frac{\partial f}{\partial x}\;\cdot \;dx\right|<\left|\frac{\partial f}{\partial y}\;\cdot \;dy\right|$	*w*_*h*_ < *w*_*r*_
Hypothesis 3	$\left|\frac{\partial f}{\partial x}\;\cdot \;dx\right|≅\left|\frac{\partial f}{\partial y}\;\cdot \;dy\right|$	*w*_*h*_ ≅ *w*_*r*_

## Materials and methods

### Participants

Twenty-seven subjects participated in the fMRI experiment (10 females, 17 males; *M* ± *SD* age = 22.48 ± 8.96 years; age range = 18–65). Eight additional subjects were excluded for the following reasons: one subject could not finish the scanning due to nausea; three subjects were excluded because their behavioral data showed no variability; and four subjects were excluded due to excessive head movement. All subjects were right-handed; had normal or corrected-to-normal vision; had no history of psychiatric, neurological, or metabolic illnesses; and were not taking medications that interfere with the performance of fMRI. Since the study involved choice (and possible consumption) of foods, all subjects were screened on arrival for any dietary restrictions such as food allergies, diabetes, or any other medical condition or religious/ethical practices that may affect choice of foods in any way.

### Stimuli

Fifty aversive food items were used in the current study. Our food stimulus set was based on the set generously shared by Plassmann et al. (2010). Some of the least aversive foods (e.g., pears; about 20% of the original list) were dropped and replaced by new items more consistently rated as unpleasant. The new items were available at local grocery stores and were chosen based on “disgust ratings” provided by a group of independent evaluators (For a complete list of foods in our stimulus set, see Table A1 in the Appendix).

The food stimuli were presented to the subjects using color pictures (72 dpi) on the computer screen during pre- and post-scanning parts, and through MRI-compatible video goggles during scanning. Stimulus presentation and response recording were implemented in Matlab, using the Psychophysics Toolbox extensions (Brainard, 1997; Pelli, 1997; Kleiner et al., 2007).

### Experiment procedure

**Figure 1:**
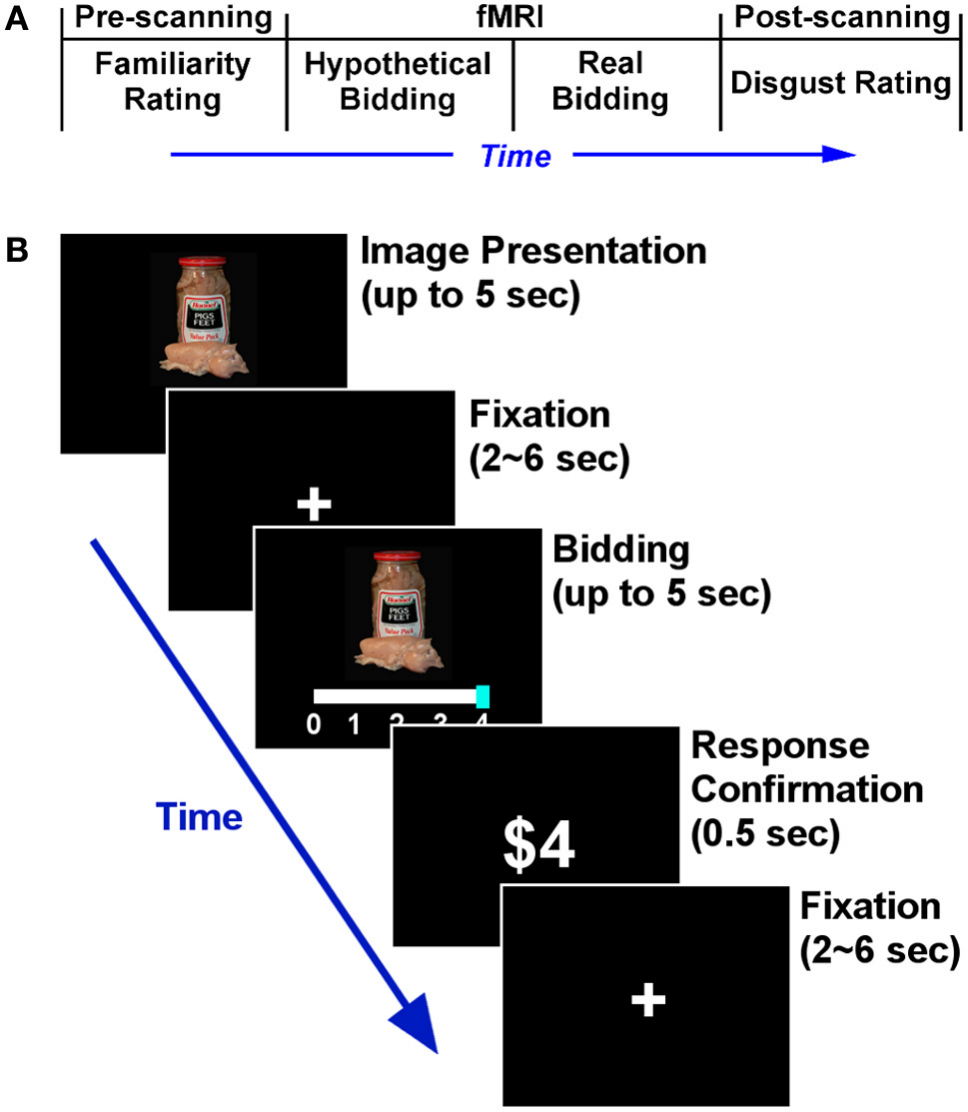
**Experimental design. (A)** Timeline of the entire experiment. **(B)** Time course of an individual trial in the scanning part. The structures of the hypothetical and real bidding trials were identical. All 50 food items were repeated across the two bidding blocks. During the food image presentation, subjects were asked to press any button as soon as they had decided how much to bid. Subjects submitted their bid using a sliding scale. The initial position of the bidding cursor (anchor) was randomized in every trial in order to avoid any potential anchoring effects (Tversky and Kahneman, 1974).

The experiment consisted of three parts—pre-scanning, scanning, and post-scanning parts (Figure 1A). At the beginning of the experiment, subjects were told that they would earn up to $50 for completing the experiment ($45 fixed plus $5 spending budget), and were informed that there were three experimental parts. Detailed instructions for each part were not given until each part began. We intentionally did *not* counter-balance the order of the hypothetical and real conditions in the scanner (following Kang et al., 2011). We discuss this design choice in the discussion.

In the pre-scanning part, subjects were shown the 50 different food images, one at a time and in random order. Subjects were asked to rate each food item on how familiar they were with it, using a scale from 0–3. The scale was defined as follows: 0 indicated “have not heard of it and have no idea of what it is” (least familiar); 1 “have not eaten the food shown, but might have heard of it and have some idea of what it is”; 2 “might not have eaten it, but have heard of it and know what it is”; and 3 “have eaten it and know what it is” (most familiar). We collected familiarity ratings for the following reason: it is possible that subjects might bid higher (not to eat) for the foods that they were less familiar with (e.g., ambiguity aversion, Hsu et al., 2005), so the familiarity rating was entered into the functional imaging data analysis to control for any potential familiarity effect on neural activity (e.g., people value familiar items more).

The scanning part had two blocks of bidding tasks, each block consisting of 50 trials—one for each food item. Both blocks were identical except that the first was hypothetical and the second block was real. Within each block, subjects were shown the same 50 food items as in the pre-scanning part, one in each trial, in random order (Figure 1B).

At the start of each scanning block, subjects were instructed as follows. They were told (to *imagine* in hypothetical block) that at the end of the experiment, one out of the 50 trials would be randomly selected by the computer and they would have to eat the food shown on that selected trial. The only way they could avoid eating the chosen food was to purchase the right not to eat it, and they had to make a bid in order to buy this right. The right to avoid the food was sold using the Becker-DeGroot-Marschak (BDM) auction mechanism (Becker et al., 1964; Plassmann et al., 2007, 2010; Kang et al., 2011). The auction worked as follows: Subjects bid one of $0, $1, $2, $3, or $4 in each trial. At the end of the experiment, the computer determined the price for the right and randomly selected one trial. Regarding the pricing, the computer would randomly generate an integer between 0, 1, 2, 3, and 4 (each integer was equally likely), and this randomly generated integer, say *p*, would determine the price of the right to avoid the food. If the bid made by the subject for a given food item, say *b*, was greater than or equal to *p* (*b* ≥ p), then the subject paid $*p* to purchase the right, and did not have to eat the item. However, if *b* < p, the subject had to eat the food shown (2–3 spoonfuls), and did not have to pay anything (see Plassmann et al. (2007, 2010) for the characteristics and limitations of the BDM auction). Note the key difference between the hypothetical and real blocks: for hypothetical trials, subjects were told to decide how much to bid while imagining that they really may have to eat the food, whereas in real trials, they knew they would have to eat the food when they did not pay enough to avoid it.

After reading the instructions for the real scanning block and prior to starting the block, subjects in the scanner gave an additional consent to actually eat the food. Subjects were made aware that they could withdraw from the experiment anytime if they did not want to continue and that in this case, they would still collect whatever they had earned up until that point. All subjects asked to eat an aversive food, at the end of the experiment, did indeed do so.

In the post-scanning part outside of the scanner, subjects were asked to rate each of the same 50 food items on how appetitive or disgusting they were to them. Ratings were entered with a sliding scale from −3 through 3 (−3: very disgusting; 0: neutral or indifferent; 3: very appetitive). This number is henceforth referred to as a “disgust rating”—note that lower values indicate a higher level of disgust.

The initial location of the anchor on the bidding scale was randomized for each trial and recorded during the scanning session. These data were used as a check for subjects' engagement in the task and possible anchoring effects. Correlations between subjects' bids and anchor positions were not significantly different from zero for most of the subjects (Table A2).

### Imaging data acquisition and preprocessing

T2^*^-weighted echo-planar images was acquired on a Siemens 3T Trio MRI scanner with a 12 channel coil (repetition time, 2030 ms; echo time, 30 ms; field of view, 192 × 192 mm; flip angle, 80°; 32 axial slices; 3 × 3 × 3 mm in-plane resolution). Images were obtained during two separate sessions of ~12 min each. To improve functional sensitivity in orbital frontal areas, each functional image was acquired in an oblique orientation of 30° off the anterior commissure-posterior commissure line (Deichmann et al., 2003). Slices were collected in an interleaved ascending manner. The first three volumes in each session were discarded to permit T1 equilibration. A high-resolution T1-weighted anatomical image (1 × 1 × 1 mm) was also acquired from each subject to facilitate localization and coregistration of functional data. All of the statistical maps reported here were rendered on the average of all subjects' structural images.

fMRI data preprocessing and analysis were performed using SPM5 (Wellcome Department of Cognitive Neurology, London, UK). Functional images were slice-time corrected, motion corrected with alignment to the first volume, spatially normalized to the standard Montreal Neurological Institute EPI template, and spatially smoothed with a Gaussian kernel (full width at half-maximum, 8 mm). Intensity normalization and high-pass temporal filtering (filter width, 128 s) were also applied to the data. The anatomical T1 images were coregistered to the mean functional EPI images for each subject and normalized using parameters derived from the EPI images. To control for nuisance effects, all regression models included six regressors indexing residual motion and two regressors for session baselines as regressors of no interest.

### Imaging data analysis

#### Primary GLM

We estimated the parameters of a general linear model (GLM) for each participant to generate voxel-wise statistical parametric maps of brain activation. The GLM assumed 1st-order autoregression and included the following regressors that capture the main events in our experiment: *H1* (hypothetical evaluation modeled as a 0-s duration event at the onset of the food image presentation in the hypothetical trials), *H2* (a parametric modulator of *H1* indicating the hypothetical bid made by the subject), *H3* (a parametric modulator of *H1* indicating familiarity rating), *H4* (a boxcar function denoting hypothetical bidding phase), *R1* (real evaluation modeled as a 0-s duration event at the onset of the food image presentation in the real trials), *R2* (a parametric modulator of *R1* indicating the real bid made by the subject), *R3* (a parametric modulator of *R1* indicating familiarity rating), and *R4* (a boxcar function denoting real bidding phase). The regressors were convolved with a canonical hemodynamic response function. The parameter estimates from this 1st-level analysis were then entered into a random effects group analysis, and linear contrasts were generated to identify regions that responded differentially to bids between the hypothetical and real conditions.

### Region-of-interest (ROI) analysis

Region of interest analyses of how activity in regions identified by the 2nd-level group analysis scaled with bids were conducted by running an additional GLM. In this analysis, trials were grouped, within each condition, according to the three levels of bids for each subject, resulting in “Low,” “Mid,” and “High” bid trials. The GLM thus had eight regressors of interest, including food image presentations with “Low,” “Mid,” and “High” bids, bidding phase for each of hypothetical and real conditions, and regressors of no interest. All regressors of interest were modeled as a boxcar function. The three levels of bids were defined as follows: for all of the subjects except for one, $0 was “Low,” $1 and $2 were “Mid,” and $3 and $4 were “High” bid; for one subject, $2 was “Low,” $3 was “Mid,” and $4 was “High” bid as this subject did not make any $0 or $1 bids in either condition. The β coefficients resulting from this *post-hoc* analysis were used to create the bar graph shown in Figures 3, 4. Note that since the ROIs were selected from the GLM described in the last paragraph, we fully expect these ROI-based analysis to provide further evidence of real-hypothetical differences. That is, the analyses are not independent. However, these GLMs do add further information that is not available from the initial GLM since they show the differences in signal change in three categories of bids.

## Results

The first results compare hypothetical and real choices, and associated response times (RTs) which provide clues about information processing. The second set of results address differences in neural activity established using fMRI, in both hypothetical and real valuations.

### Behavioral difference between hypothetical and real conditions

**Figure 2:**
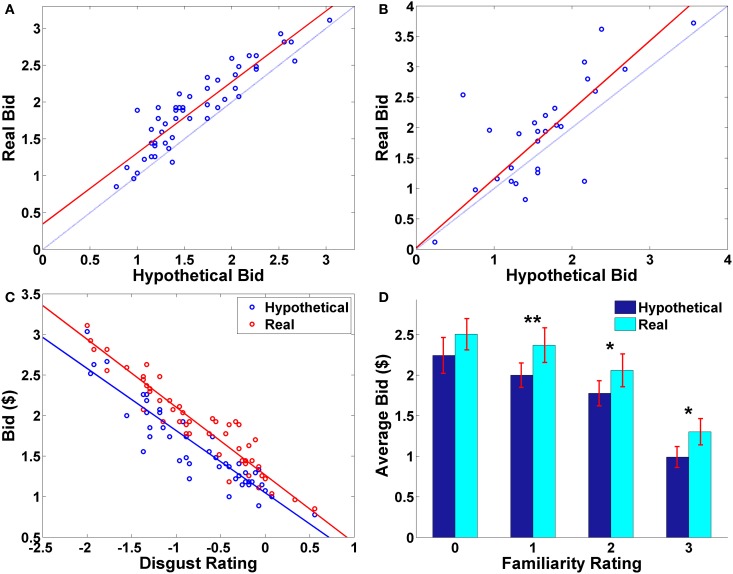
**Behavioral results. (A)** Average bid by individual food item. Each point represents an individual food item. The red solid line indicates a robust regression line (*real* bid = 0.96· *hypothetical* bid + 0.35; *p*-values for coefficients: slope < 0.0001, *intercept* = 0.0026). **(B)** Average bid by individual subject. Each point represents an individual subject. The red solid line indicates a robust regression line (*real* bid = 1.13· *hypothetical* bid + 0.03; *p*-values for coefficients: slope < 0.0001, *intercept* = 0.9207). In **(A,B)**, the translucent blue line indicates a 45° line. **(C)** Average bid as a function of average disgust rating by trial type. Each point represents an individual food item. Solid lines indicate robust regression lines (*real* bid = −0.84· *disgust* + 1.27; *hypothetical* bid = −0.77· *disgust* + 1.05; all regression coefficients significant at *p* < 0.0001). Note that on the disgust rating scale, −3 indicates “very disgusting” and 3 indicates “very appetitive”. **(D)** Average bid as a function of familiarity rating by trial type. Paired sample *t*-test, ^**^p < 0.01, ^*^p < 0.02, no asterisk = not significant.

The average hypothetical bid and the average real bid for each food were significantly correlated with each other across foods (ρ = 0.91, *p* < 0.0001). Despite this very high correlation, there are systematic differences between the hypothetical and real bids; almost all of the average real bids (averaged across subjects; *M* ± *SD* = 1.92 ±.55) were *higher* than the average hypothetical bids (*M* ± *SD* = 1.63 ± 0.53) for the same foods (Figure 2A; *t*_(49)_ = 9.22, *p* < 0.0001, paired two-sample t-test, two-sided).

Average bids by individual subject showed a similar pattern. The average hypothetical and real bids for each subject were highly correlated (ρ = 0.74, *p* < 0.0001), and the average real bid (*M* ± *SD* = 1.92 ± 0.87) was significantly greater than the average hypothetical bid (*M* ± *SD* = 1.63 ± 0.68) (*t*_(26)_ = 2.60, *p* = 0.015, paired two-sample t-test, two-sided). On average, most of the subjects made real bids that were higher than their corresponding hypothetical bids (Figure 2B).

**Figure A1:**
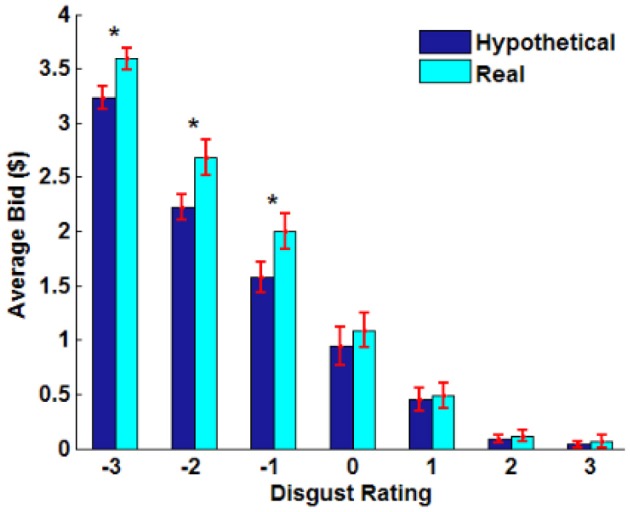
**Average bid as a function of disgust rating (−3: very disgusting; 0: neutral; 3: very appetitive) by trial type.** Paired sample *t*-test, *p < 0.02, no asterisk = not significant.

**Figure A2:**
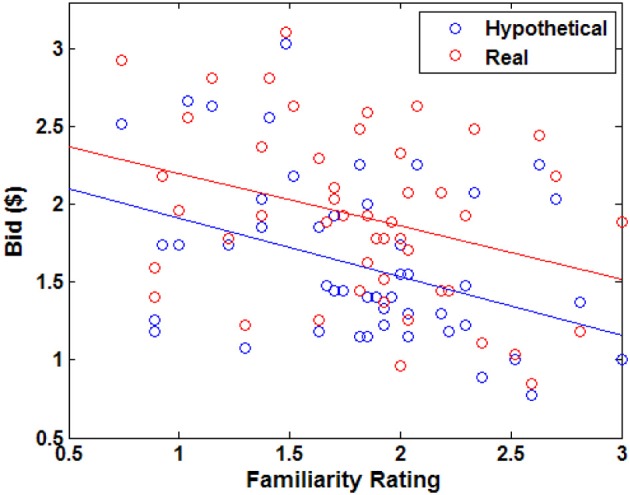
**Average bid as a function of average familiarity rating by trial type.** Each point represents an individual food item. Solid lines indicate a robust regression line; *real bid* = −0.34 · familiarity + 2.54, *hypothetical bid* = −0.38 · familiarity + 2.29 (all coefficients significant at *p* < 0.05).

Overall, subjects found the presented foods to be slightly disgusting (average disgust rating, *M*= −0.77, *SD* = 0.87) and familiar (average familiarity rating, *M* = 1.83, *SD* = 0.98). The real > hypothetical bidding difference was also shown even after controlling for disgust (Figures 2C, A1) and familiarity (Figures 2D, A2). Even including disgust and familiarity controls, real bids were $0.20 higher than hypothetical bids.

RTs during image presentation were significantly different between hypothetical and real conditions, possibly due to repeated exposure to the same stimuli (Hypothetical: *M* ± *SD* = 3.73 ± 1.41 s; Real: 3.38 ± 1.73 s; *t*_(26)_ = 2.89, *p* = 0.008, paired two-sample t-test, two-sided). In order to rule out the possibility that any neural difference between the two conditions was due to a difference in RTs, we estimated an additional GLM that was identical to the primary GLM except that RT was entered as a modulator in addition to bid and familiarity rating. However, the results from the two models did not substantially differ, so we report only the results from a simpler model without RT.

### Neural difference between hypothetical and real conditions

The GLM using a simple treatment regressor (i.e., *H*1 − *R*1) showed that there is generally more activation during hypothetical bidding as compared to real bidding (see Table A3). This is a surprising result and contrary to Kang et al. (2011), which reported more overall activity in real trials. However, since real bidding in the current study was deliberately confounded with experience (the real bids come later in the trial sequence) this deactivation could be due to either the real vs. hypothetical treatment, or to the general effect of stimulus experience and neural habituation reducing brain activity; this is a well-established effect (Thompson and Spencer, 1966; Wright et al., 2001; Fischer et al., 2003; Phan et al., 2003; Yamaguchi et al., 2004).

The more diagnostic and interesting analysis therefore focuses on regions in which activity scales with bid amounts *differentially* in hypothetical and real conditions. To find these regions we looked for areas that correlated with bids in the real trials more strongly than in the hypothetical trials. The analysis uses the contrast *R*2 − *H*2 (denoted [real × bid - hypothetical × bid] below).

**Figure 3:**
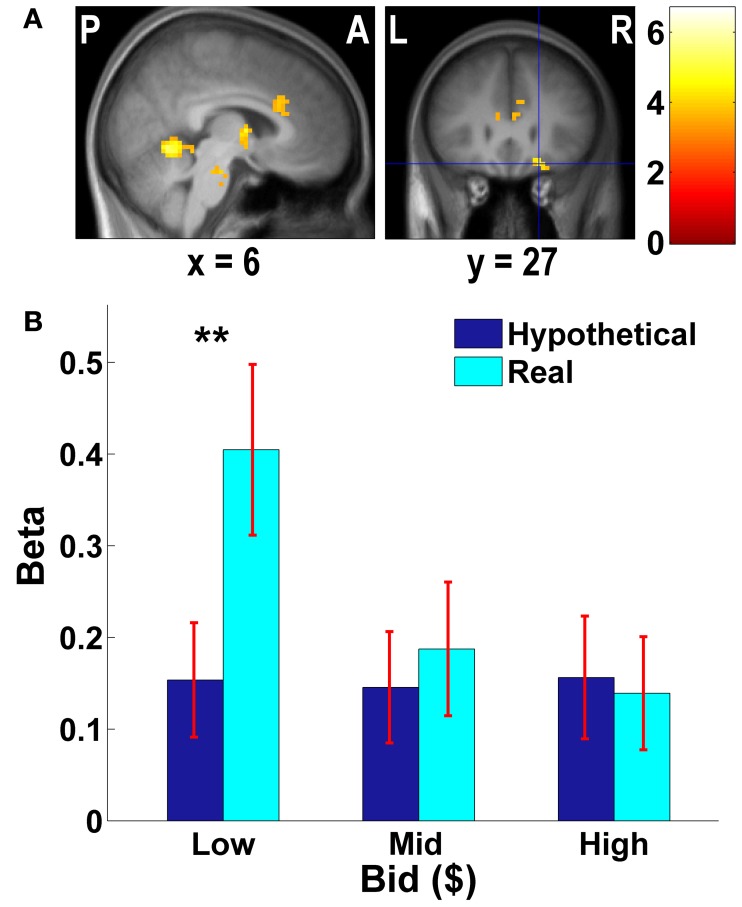
**Frontal areas exhibiting higher negative correlation with real bids than hypothetical bids. (A)** Deactivations in the [real × bid − hypothetical × bid] contrast (*p* < 0.001, uncorrected; threshold *k* ≥ 5 voxels). The color bar on the right indicates the *t*-score. **(B)** Average response in the OFC area to different levels of bids by trial type. The OFC mask was defined as a vmPFC area (peak at *x* = 21, *y* = 27, *z* = −18) identified in the [real × bid - hypothetical × bid] contrast [the area with crosshairs shown in the right panel of **(A)**]. β values extracted from this OFC mask. ^**^p = 0.016, no asterisk = not significant, paired two-sample *t*-test.

**Figure 4:**
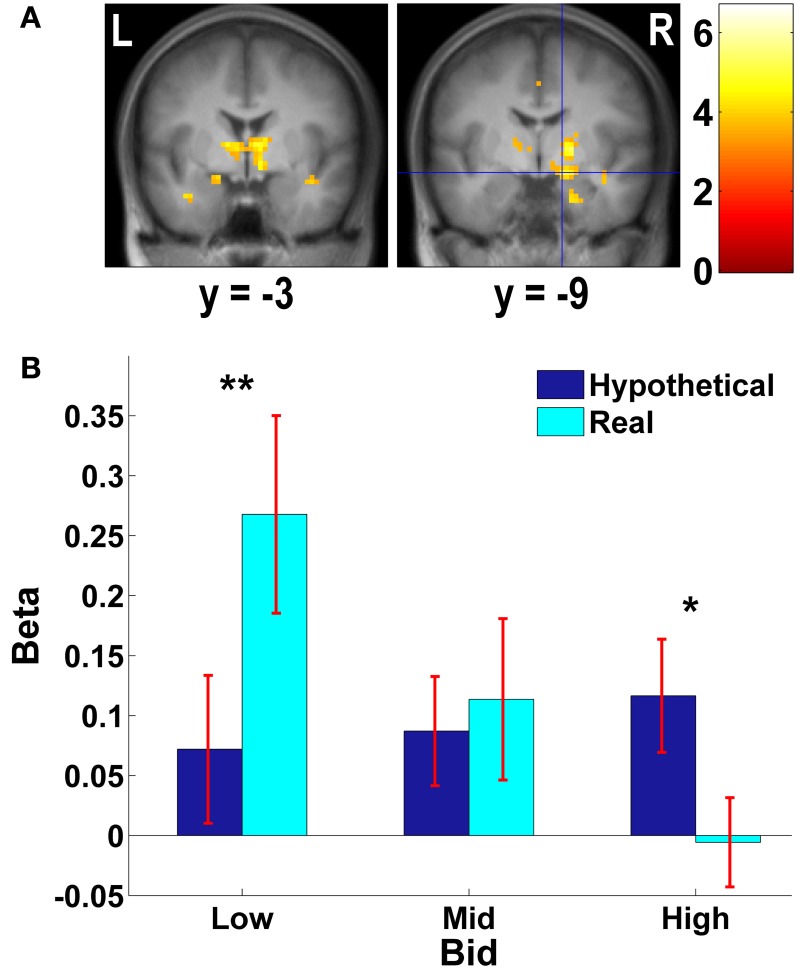
**Subcortical areas exhibiting higher negative correlation with real bids than hypothetical bids. (A)** Deactivations in the [real × bid - hypothetical × bid] contrast (*p* < 0.001, uncorrected; threshold *k* ≥ 5 voxels). The color bar on the right indicates the *t*-score. **(B)** Average response in the amygdala area to different levels of bids by trial type. Due to continuous activations in surrounding areas, the amygdala mask was defined as an intersection of the two following regions: (1) a right amygdala area (local peak at *x* = 15, *y* = −9, *z* = −12) identified in the [real × bid - hypothetical × bid] contrast [the area with crosshairs shown in the right panel of **(A)**]; and (2) a 8-mm sphere centered at the local peak of the area in (1) at *x* = 15, *y* = −9, *z* = −12. β values extracted from this amygdala mask. ^**^p = 0.015, ^*^p = 0.034, no asterisk = not significant, paired two-sample *t*-test.

**Figure A3:**
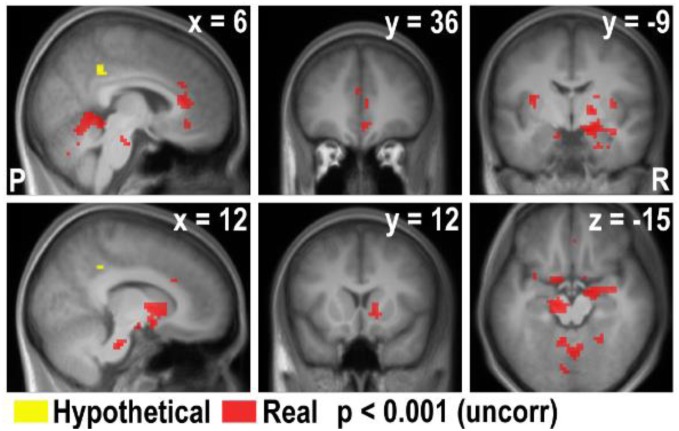
**Overlay of the areas exhibiting *negative* correlation with bids in real and hypothetical trials (overlay of the real × bid and hypothetical × bid contrasts)**.

In this analysis, there was *no* brain region identified to be more positively correlated with real bids than hypothetical bids, even at a lenient value of *p* < 0.01 (uncorrected) with a small extent threshold of 5 voxels. However, with the whole-brain analysis, we identified regions where the BOLD signal was more *negatively* correlated with real bids than hypothetical bids (Table 2, Figures 3A, 4A, and A3). These areas include the ventromedial prefrontal cortex (vmPFC), amygdala, anterior cingulate cortex (ACC), thalamus, and insula. Most of these areas, including the vmPFC, left amygdala, ACC, thalamus, and insula, are still significantly different after correction using a false-discovery rate (FDR) *p* < 0.05.

**Table 2:** **Areas showing deactivations in the difference of the parametric regressors (Real × Bid − Hypothetical × Bid)**.

**Region**	**Laterality**	**BA**	**Voxels**	**MNI coordinates**			***T***
				***x***	***y***	***z***	
Cerebellum anterior LOBE	L		178	−12	−36	−33	6.68^†^
Cerebellum	L		46	−18	−63	−27	6.51^†^
Pons	R		58	15	−27	−33	5.50^†^
Sub−lobar, thalamus, lentiform nucleus, insula	R	13	210	21	−9	3	5.32^†^
*Amygdala/hippocampus*			^*^	15	−9	−12	4.98^†^
*Ventral anterior nucleus*			^*^	6	−3	6	4.84^†^
Sub−lobar	L		101	−27	−24	6	4.72^†^
*Thalamus*			^*^	−18	−15	0	4.28^†^
***Insula***			^*^	−27	−21	15	4.08^†^
Middle occipital gyrus	R	18/19	68	27	−87	9	4.64^†^
Inferior occipital gyrus	L		51	−42	−84	−6	4.60^†^
**Middle frontal gyrus, *OFC***	R		16	21	27	−18	4.59^†^
Parahippocampal gyrus	R	35	18	33	−15	−30	4.32^†^
Sub-gyral, temporal lobe	L		5	−36	−6	−27	4.29
Anterior cingulate	R	24/32	41	12	21	30	4.19^†^
Sub-gyral, temporal lobe, superior temporal gyrus	R	21	15	39	−6	−18	4.09^†^
**Amygdala**	L		7	−18	−3	−15	4.05
Superior temporal gyrus	L	38	9	−36	6	−21	3.92
Anterior cingulate	L	9/32	13	−12	30	15	3.90
Cingulate gyrus	R		5	0	−15	42	3.73

*Height threshold: t_(26)_ = 3.67, p < 0.001 (uncorrected); extent threshold, k = 5 voxels. L, left; R, right*.

† Survives whole-brain FDR correction at p < 0.05;

* *part of a larger cluster*.

We previously found that for appetitive goods (consumer products), the vmPFC is more strongly involved in valuation in real decision making compared to hypothetical decision making (Kang et al., 2011). That previous study, using appetitive goods, did *not* find the amygdala to be involved in valuation. However, the amygdala is thought to play a key role in processing of aversive stimuli and aversive conditioning (among other functions) (Whalen, 1998; Phelps, 2006; Johansen et al., 2010). Hence, we further explored how bids appeared to be encoded in both vmPFC and amygdala areas.

As Figures 3B, 4B show, the vmPFC and the amygdala areas show a significant negative linear trend across different levels of bids in real trials only; such a trend is not observed in hypothetical trials. That is, less aversive goods (which subjects pay less to avoid eating) activate the vmPFC and the amygdala more strongly than more aversive goods, but only in real trials.

**Figure A4:**
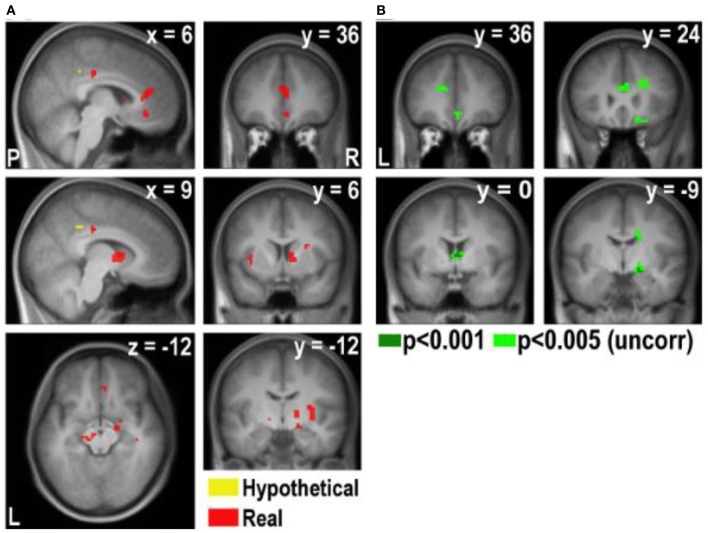
**Areas exhibiting higher correlation with disgust rating in real and hypothetical trials (note that a lower disgust rating indicates a higher level of disgust). (A)** Overlay of the hypothetical × disgust and real × disgust contrasts, *p* < 0.001 (uncorrected). **(B)** [real × disgust - hypothetical × disgust] contrast.

When the food disgust rating was used in place of dollar bids, similar regions of brain activity are found (Figure A4). This finding is important because it implies that economic valuation, per se, is not fundamentally different than judgments of disgust, at least for these types of aversive foods.

**Figure A5:**
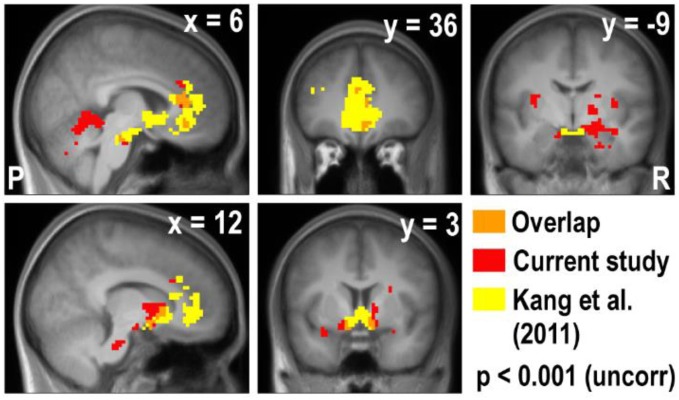
**Overlay of the deactivation map of the real × bid contrast and the activation map of the real × mDV contrast in Kang et al. (2011).** The vmPFC, ACC, and striatum appear in both studies, but the amygdala only appears in the current study.

Lastly, we compared the areas that were parametrically modulated by bids in the current study and with the areas modulated by decision values of appetitive goods in the previous study by Kang et al. (2011). Due to the lack of deactivation in the Hypothetical × Bid contrast in the current study, we overlaid the areas that negatively correlated with *real* bid in the current study and the areas that were positively correlated with a *real* decision value of appetitive goods in the previous study (Figure A5). We found that the vmPFC, ACC, and ventral striatum (VStr) appeared in both studies, but the amygdala and the surrounding areas only appear in the current study of the aversive domain.

## Discussion

This study is the first to compare the willingness-to-pay to avoid aversive consumption outcomes (unpleasant foods), in the two conditions of non-binding hypothetical decision and binding real decision. Previous studies with appetitive stimuli typically find that hypothetical valuations are higher than real valuations. We find the opposite result. Binding, real bids to avoid eating unpleasant foods were larger than hypothetical bids. The within-subject design provides good statistical power to show that the real > hypothetical bias is highly significant across both foods and subjects.

Before proceeding, it is useful at this point to squarely address potential threats to the validity of our scientific inference from the deliberate design choice to elicit both hypothetical and real valuations of the same foods in a fixed order (i.e., two exposures per food, hypothetical then real). We fixed this order out of concern that eliciting real bids first would lead to a mental state for the second hypothetical block fundamentally different than that in most lifelike situations where hypothetical judgments are made.

Many studies have presented the same stimuli multiple times (e.g., Fitzgerald et al., 2009; Hare et al., 2009), and found consistent signals. The biggest threat to validity when stimuli are judged repeatedly is that the repeated judgments are artificially consistent. However, any such effect could lower the capacity of the design to detect a highly significant hypothetical < real difference in willingness-to-pay; and yet, we do find such a difference. (Therefore, it is likely that a between-subjects design could show a much larger difference, both neurally and behaviorally; FeldmanHall et al., 2012b used a between-subjects design and found striking differences.) Furthermore, the biggest concern with neural activity would be possible habituation of a neural signal. If there was habituation of neural activity to the foods over time, we would expect the neural activity to diminish to the baseline activity or (perhaps) be less value-sensitive for the real trials that come after the hypothetical ones. However, we found just the opposite, that is, stronger sensitivity of neural activity to the value, which is not consistent with the possibility of habituation. And behavioral experiments using appetitive goods by Kang et al. (2011) showed that there were real vs. hypothetical differences in both possible treatment orders. Thus, we argue that the potential risks from the within-subject design with fixed treatment order are not strongly evident in the data. In addition, there are many statistical benefits of repeating the same stimuli in the hypothetical and real conditions. Doing so controls for nuisance variables such as physical and psychological aspects of stimuli (e.g., color, shape, experience, memory) that might be correlated with stimulus value, but are not involved in the valuation of aversive stimuli per se.

Returning to the scientific contributions of the current study, the combination of results herein with earlier results from Kang et al. (2011) (and many earlier behavioral studies showing positive hypothetical bias) rejects the hypothesis that real dollar valuations are lower in general, i.e., for both appetitive goods and aversive *bads*.

While this conclusion is tentative, it is important because a popular theory about hypothetical bias is that people underestimate the value of money when expressing hypothetical values. An example is the influential report (Arrow et al., 1993) by a panel of academic economists on how to best elicit and use “contingent valuation” survey measures to establish reasonable prices for non-market-traded goods and services (such as clean air). They specifically “emphasize[d] the urgency of studying the sensitivity of willingness to pay responses to… reminders of other things on which respondents could spend their money.” The panel's conclusion followed from a conjecture that opportunity cost reminders would *lower* hypothetical responses because disutility from monetary payment is underestimated in hypothetical choice, so that reminders would lead to better approximate real values (as shown, in fact, by Knoepfle et al., 2009).

Our results do *not* support the idea of a general strong devaluation of money during hypothetical valuation (for both appetitive and aversive objects). Instead, the results lend tentative support to a different hypothesis (hypothesis 2) mentioned in the introduction: aversive experience of visceral factors (i.e., disgust) is more strongly underestimated in hypothetical choice than aversive experience of more cognitive factors (i.e., paying money) because people in an affectively “cold” state easily fail to appreciate the influence of a “hot” visceral factor that is not currently experienced upon their preference and behavior (Loewenstein, 1996, 2000, 2005). We call this the “visceral response underestimation” hypothesis.

In valuing appetitive goods hypothetically, “overly optimistic consumers” (Tanner and Carlson, 2009) would probably overestimate benefit from consumptions of goods and to underestimate the pain of paying, leading to hypothetical values that are too high. In valuing aversive goods hypothetically, disutilities from both of spending money and eating unpleasant foods are generally expected to be underestimated. However, disgust of eating unpleasant foods is likely to be stronger in real choice if there is a tendency to more strongly underestimate the influence of the more visceral factor. Our behavioral finding (higher WTP in real choice) is consistent with this account.

The brain imaging results reported also support this account. Stronger encoding of “better” valuation (i.e., lower bids to avoid less aversive foods) during real choice is found in cortical regions that are well-established to encode value (vmPFC, ACC, and VStr). Notably, Plassmann et al. (2007, 2010) find that the vmPFC encodes both increased value for appetitive goods, and decreased distaste (a positive improvement) for aversive goods. Tom et al. (2007) found a similar common encoding in the VStr and vmPFC for both increased potential money gains and decreased money losses.

Most importantly, we find more value-sensitive activity during real choice in the insula, amygdala, and hippocampus. The insula is thought to encode general emotional and visceral discomfort (Craig, 2002, 2009), ingestive disgust (Harrison et al., 2010), risk (Preuschoff et al., 2008; Mohr et al., 2010), and empathy for pain (Singer et al., 2004, 2009; Bernhardt and Singer, 2013). The amygdala responds rapidly to impending threat, creating neural vigilance (among other functions) (Adolphs et al., 1998; Whalen, 1998). The visceral response underestimation hypothesis is consistent with these functional attributions, assuming that anticipation of actually eating unpleasant foods is viscerally uncomfortable or threatening, as compared to merely imagining so as in hypothetical choice.

Keep in mind that insula and amygdala activity is *stronger* for low-bid (i.e., less aversive) bad foods when choices are real. This direction is also consistent with Plassman et al.'s (2010) finding, that parahippocampal and insula activity is higher response to lower bids to avoid unpleasant foods. Nonetheless, we would not have been surprised by the opposite pattern—i.e., a stronger emotional reaction to the worst foods that people would pay the most to avoid eating. Given our somewhat surprising result, we offer a speculative reverse inference about why the least-bad foods generated the most insula and amygdala activity in real choice (compared to hypothetical). The signal changes displayed in Figure 4B seem to offer a clue. Note that in hypothetical choice, bads which receive higher bids to avoid have higher amygdala activity. Thus, in this “baseline” condition worse foods generate more amygdala reaction, as one might expect if amygdala is reflecting vigilance or negative emotion. However, when the real condition takes place, subjects now think about the prospect of actually eating the foods. If they bid high they won't have to eat the food, and the amygdala reaction to the worst (high-bid) foods is actually much lower than in the hypothetical condition. Oppositely, the least bad (low-bid) foods are ones they are likely to eat—but in the real condition only—since they bid low. We speculate that the heightened amygdala activity encodes a reaction not to the stimulus per se, but to the expected displeasure of having to worry about “really” eating it. That is, part of the switch from hypothetical to real evaluation is a switch from a subjective emotional reaction to the food itself (in the hypothetical case), to a reaction to the prospect of whether the food will have to truly be eaten (in the real, low-bid case).

A way to test this hypothesis is to immediately display the outcome of the BDM mechanism that determines whether the food will be eaten or not, given their bid. If a person bids 3, for instance, but the BDM draw if 4, then they did not bid enough and will have to eat the food. If our reverse inference is correct, then when they are surprised at having to eat the food, even after bidding so high, we will see a strong aversive signal in amygdala (similar, perhaps, to the hypothetical-condition signal.

This fMRI evidence of differential insula and amygdala activity is new evidence of a biological encoding of a hot (real choice)-cold (hypothetical choice) empathy gap, in the brain. FeldmanHall et al. (2012b) also find more activity in amygdala during real moral choice, compared to imagined hypothetical choice, and in temporal-parietal junction (TPJ) and right insula. The convergent evidence of stronger activity in amygdala and insula in both studies, in different domains of unpleasant choices (food and pain administration) is encouraging, and invites further studies to establish further robustness.

Speculation about visceral response underestimation also suggests some potential ways to “de-bias” hypothetical choices for future research. For example, it is known that the amygdala responds to fearful or threatening stimuli such as a fearful face (and even fearful eye whites only) (Whalen et al., 1998, 2004) and electric shocks (Phelps et al., 2001). Insula also responds reliably to exogeneous stimuli that are unpleasant. Hence, one future direction is to manipulate amygdala or insula activity by using such stimuli during hypothetical choice. The idea is that stimulation of such regions by external stimuli might “fool” the neural circuitry into making judgments as if it is in a hot state (as in classic arousal misattribution, e.g., Dutton and Aron, 1974). Inducing an artificial hot state could produce hypothetical choices that are better forecasts of actual real choices. Another direction is to tap visceral urges during hypothetical valuation by having subjects inspect and smell real aversive food items, facilitating them to more easily integrate visceral factors into their hypothetical valuations. A third direction is to manipulate the degree of imagination exerted in the hypothetical case [an approach often used in economic surveys and also in FeldmanHall et al. (2012a)] and see how such treatments alter brain activity.

Note that in our study hypothetical bids are (significantly different but) highly correlated with real bids, and that both types of bids are also significantly correlated with disgust ratings. However, despite such high correlation between the two behavioral measures, there is much less neural activity during hypothetical valuation than real valuation (Figures A3, A4). For many purposes, knowing that real and hypothetical behavior covaries strongly across stimuli is good enough to justify relying on hypothetical choice as a guide (especially when real choices are unethical, expensive, or otherwise problematic). However, the weak neural response to bids in the hypothetical condition suggests that researchers in social and consumer neuroscience who want to understand real choices neurally could find that purely hypothetical contexts generate weaker signals, and that making choices real will often add statistical power (and fidelity). There has been growing academic interest in applying machine learning techniques to neuroimaging data in order to predict purchase choices, using either a real choice or a hypothetical choice paradigm (Grosenick et al., 2008; Tusche et al., 2010; Smith et al., 2012). Although such effort has been somewhat successful (in terms of accuracy rates), the findings of the current study suggests that predictive models estimated using hypothetical data are limited in how well they can accurately predict real purchase choice by how different the underlying neural circuitries are.

Finally, we note that many data about how people evaluate aversive experiences are inherently hypothetical, rather than real, particularly if the data are collected to pre-value or anticipate potential averse events. An important domain is medical decision making. Patients often read hypothetical scenarios regarding different stages of a disease and treatment toxicity in order to make decisions about treatments (e.g., with end-stage cancer, choice between chemotherapy that could extend life by 4 months with severe side effects versus supportive care that could only alleviate symptoms) (O'Connor et al., 1987; Malenka et al., 1993; Yellen et al., 1994; Silvestri et al., 1998; Gurmankin et al., 2002; Levy and Baron, 2005). Further, a physician, who is giving medical recommendations, may need to put herself in the hypothetical situation of being in her patients' minds (particularly for a minor or an incapacitated patient). Cancer patients often say that they would not want to receive grueling cancer treatments (e.g., chemotherapy) but then change their minds when they do get cancer (Loewenstein, 2005). Further understanding of how the brain makes both hot and cold (real and hypothetical)decisions could guide people and societies to make these difficult decisions more effectively.

## Author contributions

Design: Min J. Kang, Colin F. Camerer. Data collection: Min J. Kang. Data analysis: Min J. Kang. Writing: Min J. Kang, Colin F. Camerer.

### Conflict of interest statement

The authors declare that the research was conducted in the absence of any commercial or financial relationships that could be construed as a potential conflict of interest.

## Footnotes

^1^A referee pointed out that the pain of paying may be quite low in our scenario, since the payments are only $0–4 out of a $50 endowment. The hypothetical bias (and perhaps associated neural activity) could be reversed if the dollar scale was much larger and the aversive bad was less viscerally unpleasant, or mentally remote, as in a case like buying insurance.

## Abbreviations

WTP: willingness-to-pay
CS: consumer surplus.

## Appendix

**Table A1:** **List of 50 aversive food items used in the study**.

**Product name**	**Product name**
Anchovies	Mock duck meat
Artichoke	Mustard
Baby food (carrot)	Onions
Baby food (chicken and rice)	Oyster sauce
Baby food (green beans)	Oysters
Baby food (lamb)	Palms
Baby food (mac and cheese)	Pickled jalapeno peppers
Baby food (squash)	Pigs feet
Baby food (vegetables and beef)	Plums
Baby clams	Plum sauce
Beets	Pork rind
Butter	Relish
Capers	Rice vinegar
Chicken spread	Sardines
Clam juice	Sauerkraut
Clams	Sesame chili oil
Corned beef	Shrimp sauce
Fish sauce	Spam bacon
Ginger crisp	Spam spread
Graduate chicken sticks	Spinach
Ham spread	Squid
Horseradish	Tabasco
Liver pate	Thai green curry paste
Liver wurst spread	Wasabi
Mackerel	Wax beans

*Most of the food items were canned or bottled, and the remainder of them (e.g., butter or wasabi) were packaged in a small box or container. Hence, all foods items were stored easily in the lab or the nearby office space*.

**Table A2:** **Correlation between bids and anchors**.

	**Correlation**	
**Subject ID**	**Hypothetical**	**Real**
1	0.007	0.007
2	0.316^*^	−0.213
3	−0.018	0.063
4	−0.204	−0.022
5	−0.184	0.020
6	0.068	−0.068
7	−0.041	0.187
8	0.085	0.339^**^
9	0.072	−0.262
10	0.002	0.082
11	−0.159	0.050
12	0.104	0.018
13	−0.176	−0.010
14	0.078	0.035
15	−0.152	−0.228
16	0.410^**^	0.127
17	−0.037	−0.075
18	−0.053	0.136
19	0.185	0.109
20	0.129	0.005
21	0.183	0.036
22	−0.102	−0.062
23	0.150	0.027
24	−0.024	−0.110
25	−0.176	−0.130
26	0.141	0.026
27	0.166	−0.030
Mean	0.028	0.002
Std Err	0.030	0.025

*The average correlations for hypothetical and real trials are not significantly different (paired sample t-test, p = 0.471)*.

* p < 0.05,

** *p < 0.01*.

**Table A3:** **Areas showing activation in the contrast of hypothetical vs. real evaluation, identified by the whole-brain analysis [(hypothetical − real) contrast]**.

**Region**	**Laterality**	**BA**	**Number of voxels**	**MNI coordinates**			***t***
				***x***	***y***	***z***	
Caudate, sub-lobar	L		40	−18	6	18	4.85
Superior temporal gyrus	R		64	39	−45	3	4.42
Posterior cingulate	L	31	75	−6	−57	12	4.39
Lentiform nucleus, putamen	L		24	−27	−21	0	4.18
Inferior frontal gyrus	R		33	33	33	9	4.16
Superior frontal gyrus	R	8	15	18	48	51	4.06
Sub-lobar, amygdala	L		48	−9	−6	−9	3.94
***Amygdala***			^*^	−21	0	−15	3.19
Medial frontal gyrus	L	10/11	129	−3	57	21	3.85
***Medial frontal OFC***			^*^	3	57	−6	3.15
Corpus callosum	R		21	12	30	15	3.76
Middle temporal gyrus	L		26	−60	−15	−15	3.72
Posterior cingulate	R		51	15	−48	9	3.67
*Parahippocampal gyrus*			^*^	9	−39	−3	3.14
**Medial frontal gyrus, mOFC**	L	11	16	−3	36	−18	3.35
Inferior frontal gyrus	L		31	−48	24	−6	3.34
*Lateral OFC*			^*^	−42	27	−15	3.31

*Height threshold: t_(26)_ = 2.78, p < 0.005 (uncorrected); extent threshold, k = 15 voxels. L, left; R, right*.

* *Part of a larger cluster*.
